# Book Reviews

**Published:** 1830-11

**Authors:** 


### BIBLIOGRAPHICAL NOTICES.

*A few Words on Quackery.* By DEMOCRITUS JUNIOR. Burgess and Hill, 1830.

WE lament that the author of these "few words" has not entered more at length into the subject of quackery, although he has done enough to show that he has the rare talent of being severely sarcastic, without incurring the censure of vulgar personalities. We trust we shall hear more from Democritus Junior; he wields his pen with too much dexterity to allow it to remain idle. The motto he has selected from Pope is happy:

> "Plain truth, dear St. John, asks no flowers of speech."

His principal object is to show the why and the wherefore of the success with which Messrs. Goss and Co., Mr. St. John Long, and other "gapers after fame and money," have been so fortunate as to meet.

A fidgetty, fretful girl, whose parents lack wit, but not money, thinks it well to be genteelly consumptive: she *must* go to Mr. Long. "Now the truth is, that Miss Louisa's complaints are simply nervous or hysterical; and any thing capable of changing the hypochondriac current of her ideas, or relieving the dull monotony of her existence, will effect a temporary cure, be the remedy agreeable or disagreeable, be it prescribed by Maton, or inflicted by Long: thus marriage, a trip to Brighton, or a legacy of 10,000*l.* will prove as effective as asafœtida, a residence in a disturbed Irish district, reading Mr. Long's book, or being flayed by his liniment."

"You see, my dear public, I have no spite against the famous liniment; quite the reverse: I believe it to be a good, scorching, burning, skin-destroying, slough-creating liniment, as one might wish to see on a summer's day, as good a liniment as the healing god might have used, when whilome in Harley street, (I beg pardon, in Phrygia,) he cured Marsyas of all his ailments in one single visit. Nay, more, I must freely confess that there is one quality in which this vigorous liniment

——'stands alone,
Like Adam's recollection of his fall;'

a quality to which a red-hot poker, and sulphuric acid, and the cat-o'-nine-tails, and caustic potash, and other of its elder-born brothers, make no pretension; I mean its acute discriminating power. This is not one of those vulgar embrocations that fasten on friend and foe alike; one of those hot-headed inconsiderate lotions that would treat the skin behind which the lungs of Stentor were entrenched, like the skin of a Hawkes or a Fennel. No, no; a liniment educated in the polite air of Harley street would scorn the sordid manners of its relations brought up in hospitals, whose unfashionable site I am almost ashamed to mention. This sharp and superhuman discernment will, certainly, place the liniment far above the stethoscope; no man will content himself with looking, or rather hearing, when he can look and cure at the same time."

"How curious it must be to observe a broad excoriation, the telegraphic signal of some vast abscess within, and to contrast it with the minor and interrupted flayings that indicate the presence of tubercles!" The young lady, who fills up her leisure by fancying herself consumptive, and thus occupies her vacant mind, "after paying twenty or thirty visits (and twenty or thirty guineas), is compassionately dismissed cured, or greatly relieved, with some skin, a little money, and the most perfect faith in the wonder-working embrocation. So astonishing a recovery as this spreads along the Surrey road: Clapham is amazed, Balham Hill pricks up its ears, and Tooting thinks that the good St. John deals with something that is not right. However, it all brings grist to the mill. Surrey carriages are seen clustering at the door of the arch-skinner: Surrey sovereigns jingle in his pocket. Miss Epsom has sprained her foot at the races; it is rubbed, and is well. The right honourable Lord Leatherhead feels dull and stupid; he is for a time tortured into liveliness."

We verily believe that such are the kind of cases upon which the reputation of Mr. Long is founded. Other medicasters have been equally successful, and have also been "warmed in the sun of aristocratic praise." Thus says Democritus: "Mrs. Stephens was the St. John Long of 1738-42; perhaps even longer. I confess it is an injustice to her memory to mention them together. She enjoyed the praises of Hales and Hartley: Mr. Long is too happy to be mentioned by the Literary Gazette."

Democritus Junior is evidently a man of liberal education: his wit is classical, and the style of the little *jeu d'esprit* with which he has favored us very well adapted to the object in view, of exposing ignorance and imposition, and of ridiculing the little follies of the great.

---

*An Outline of the Sciences of Heat and Electricity.* By Thos. Thomson, m.d. Regius Professor of Chemistry in the University of Glasgow, f.r.s. &c. 8vo. pp. 583. Baldwin and Cradock, 1830.

Although we cannot enter into a copious analysis of this work, it must not be inferred that we are insensible of the philosophical importance of the subjects it discusses, or of the ability with which the learned author has treated them. Students, who are desirous of obtaining information upon the subjects of heat and electricity, will need no other assistance than the present volume, which contains an abridgement of the lectures on those subjects which are annually delivered by the author in the College of Glasgow, as a necessary introduction to the elements of chemistry. As a text-book for Dr. Thomson's pupils, this work must be particularly valuable.

### MONTHLY LIST OF MEDICAL BOOKS.

